# Retained Menses from Imperforate Hymen

**Published:** 1873-02

**Authors:** A. Ford

**Affiliations:** Bowne, Kent County, Michigan


ARTICLE IV.—*Retained Menses from Imperforate Hymen.* By A. FORD, M.D., Bowne, Kent County, Michigan.

About six months ago I was called to see Miss H., aged 14 years. Found her suffering severely with pain in the back and through the lower part of the abdomen; complained of a lump in the region of the navel. On examination, found a hard, movable tumor reaching as high as the umbilicus. Patient quite weak, hardly able to stand. Questioned the mother in reference to the appearance of the menses, who said that she thought it time for the menses to appear; that for about two years the girl had had regular periods of being unwell, but no discharge had taken place. I diagnosed retained menses with imperforate hymen, or closure of the mouth of the uterus; and obtained permission to make a digital examination. Found the hymen tense as a drum-head, and bulging through the vulva. I made an incision with a curved bistourie, and the retained menstrual fluid gushed out. It was dark colored, thick, and almost ropy; there was a large quantity, but do not know how much. When the discharge ceased, she said, "Mother, the pain is all gone!" She has been *regular* ever since, and is as fresh and healthy a girl as you would wish to see.

